# Supplementary figures and images for: Identification and molecular characterization of the alternative spliced variants of beta carbonic anhydrase 1 (βCA1) from Arabidopsis thaliana
Source: PeerJ. 2021 Dec 23;9:e12673. doi: 10.7717/peerj.12673 (PMC8710251; doi:10.7717/peerj.12673)

AT3G01500

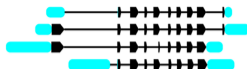

Potri.001G348900

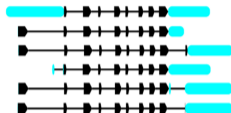

Pp3c1\_19190

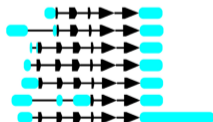

Glyma.19G007700

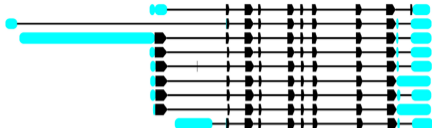

Sobic.003G234500

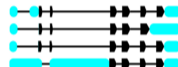

Bradi2g44856

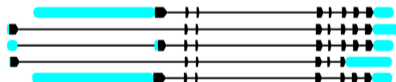

LOC\_Os01g45274

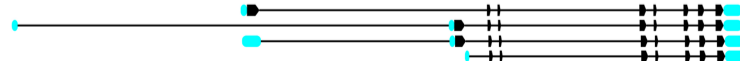

Supplement: Supplemental Information 1 — AT3G01500 from Arabidopsis thaliana, Glyma.19G007700 from Glycine Max, Potri.001G348900 from Populus trichocarpa, Sobic.003G234500 from Sorghum bicolor, Pp3c1_19190 from Physcomitrella patens, Bradi2g44856 from Brachypodium distachyon, and LOC_Os01g45274 from Oryza sativa were shown. The black boxes represent exons, solid lines represent introns, and bright blue boxes represent untranslated regions (UTRs). [file peerj-09-12673-s001.pdf]

**A**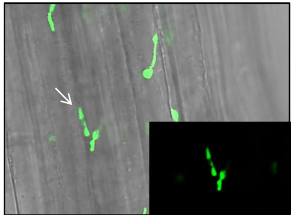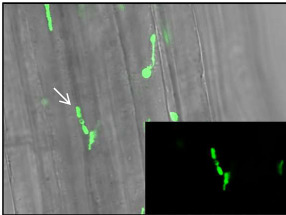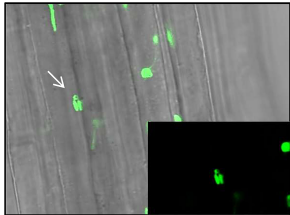**B**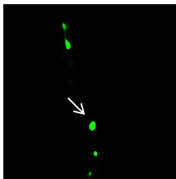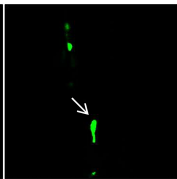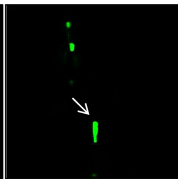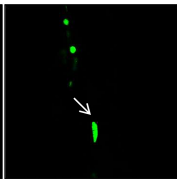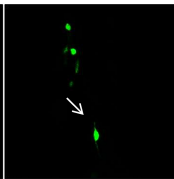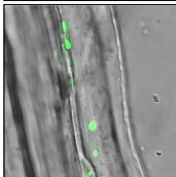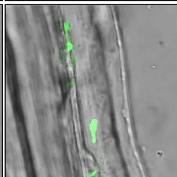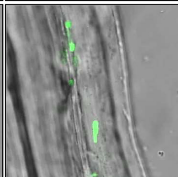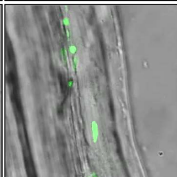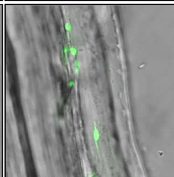

Supplement: Supplemental Information 2 — (A–B) dynamic GFP signals were observed from the mature zone of transgenic plants (βCA1.1-GFP, βCA1.2-GFP and βCA1.3-GFP). [file peerj-09-12673-s002.pdf]

**Seedling**      **Shoot**      **Root**

**CA1.1-GFP**

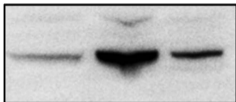

**CA1.2-GFP**

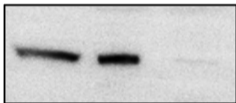

**CA1.3-GFP**

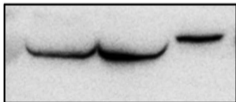

**CA1.4-GFP**

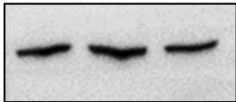

**$\alpha$ -GFP**

Supplement: Supplemental Information 3 — The protein accumulation of four βCA1 transcripts was analyzed by western blotting. [file peerj-09-12673-s003.pdf]

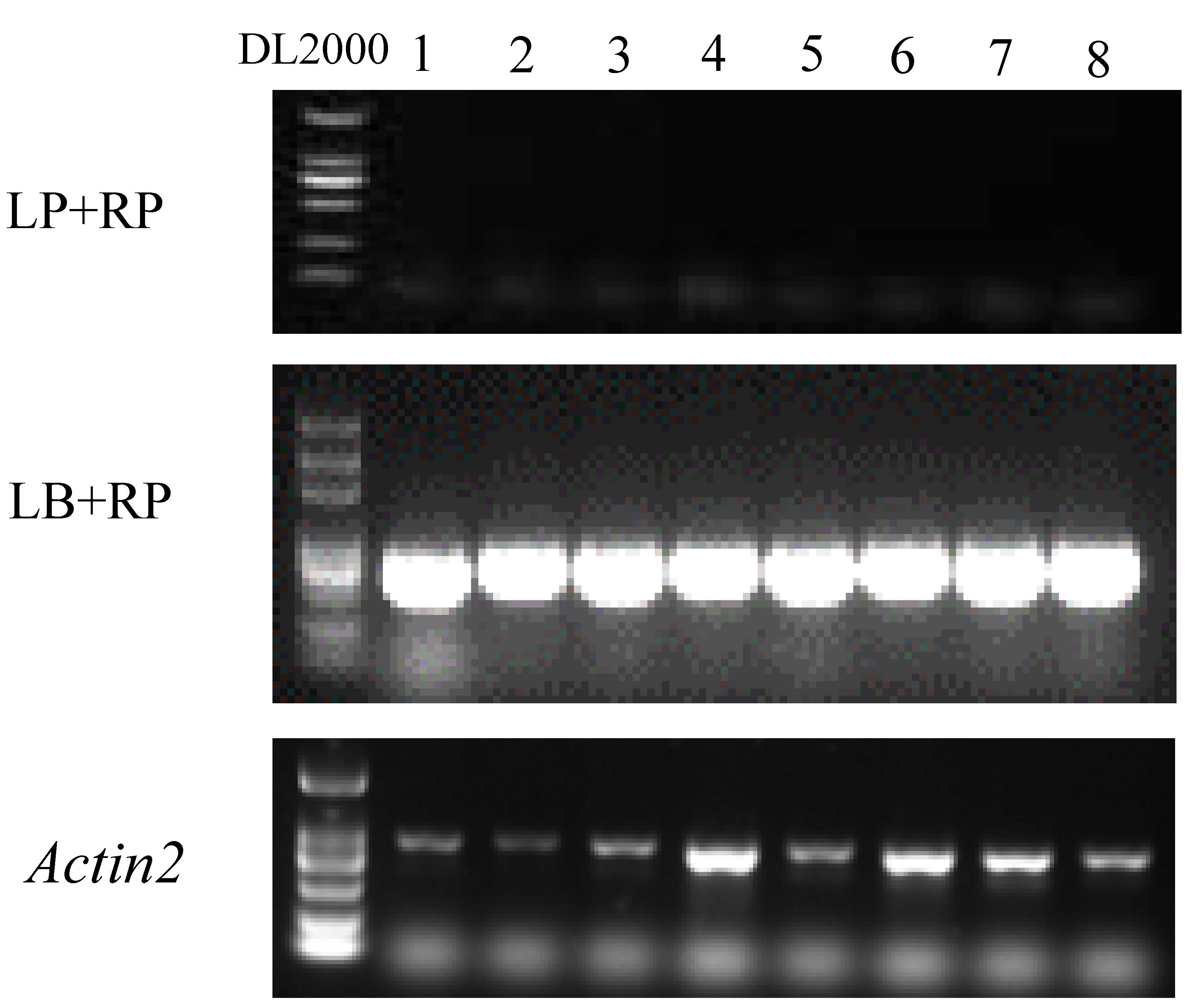

Supplement: Supplemental Information 4 — 1-8: individual Arabidopsis seedlings, DL2000 was for the DNA ladder. Actin2 was used as the control. [file peerj-09-12673-s004.jpg]

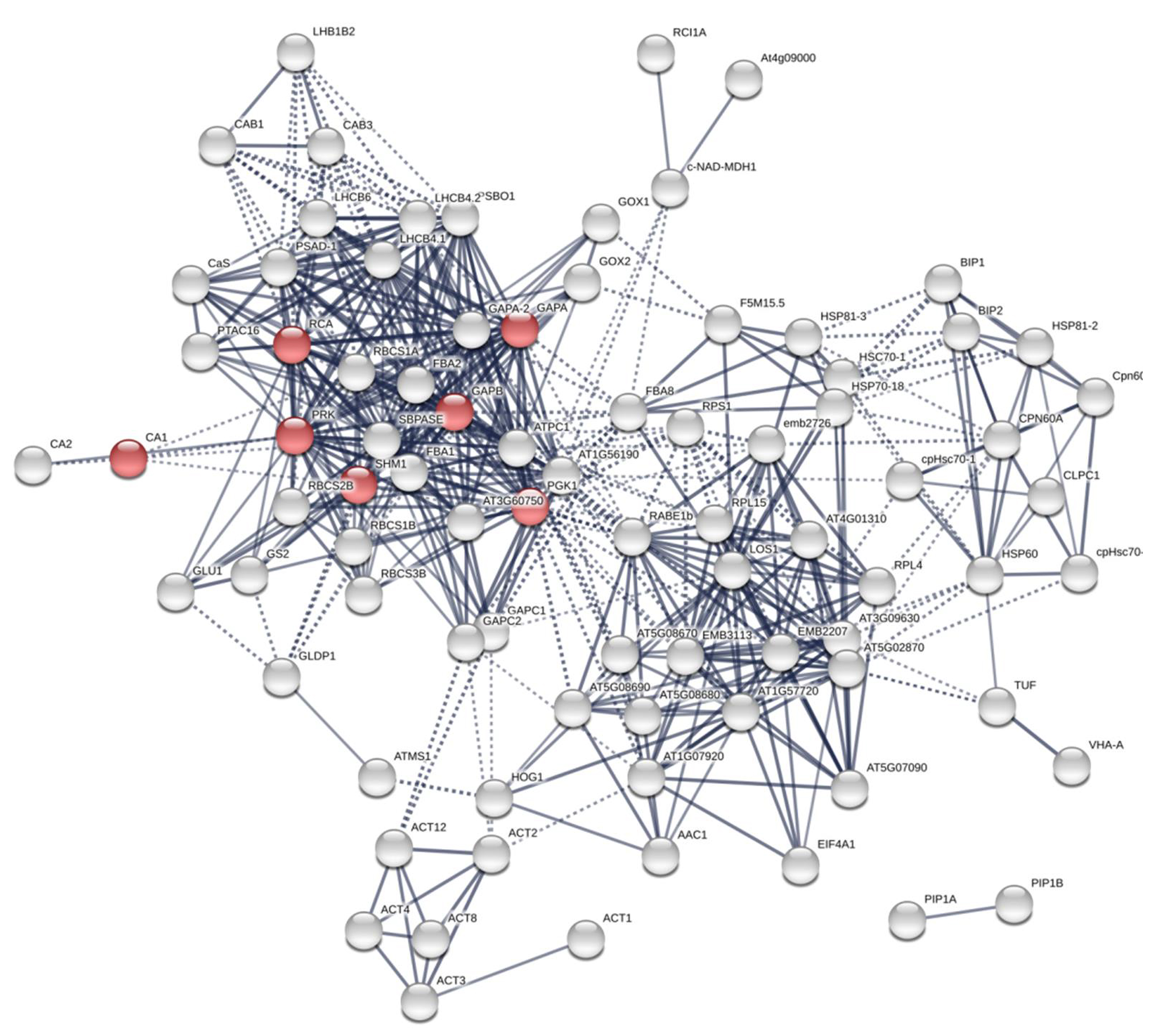

Supplement: Supplemental Information 5 — The proteins with localization predicted in stromule were in red. [file peerj-09-12673-s005.jpg]

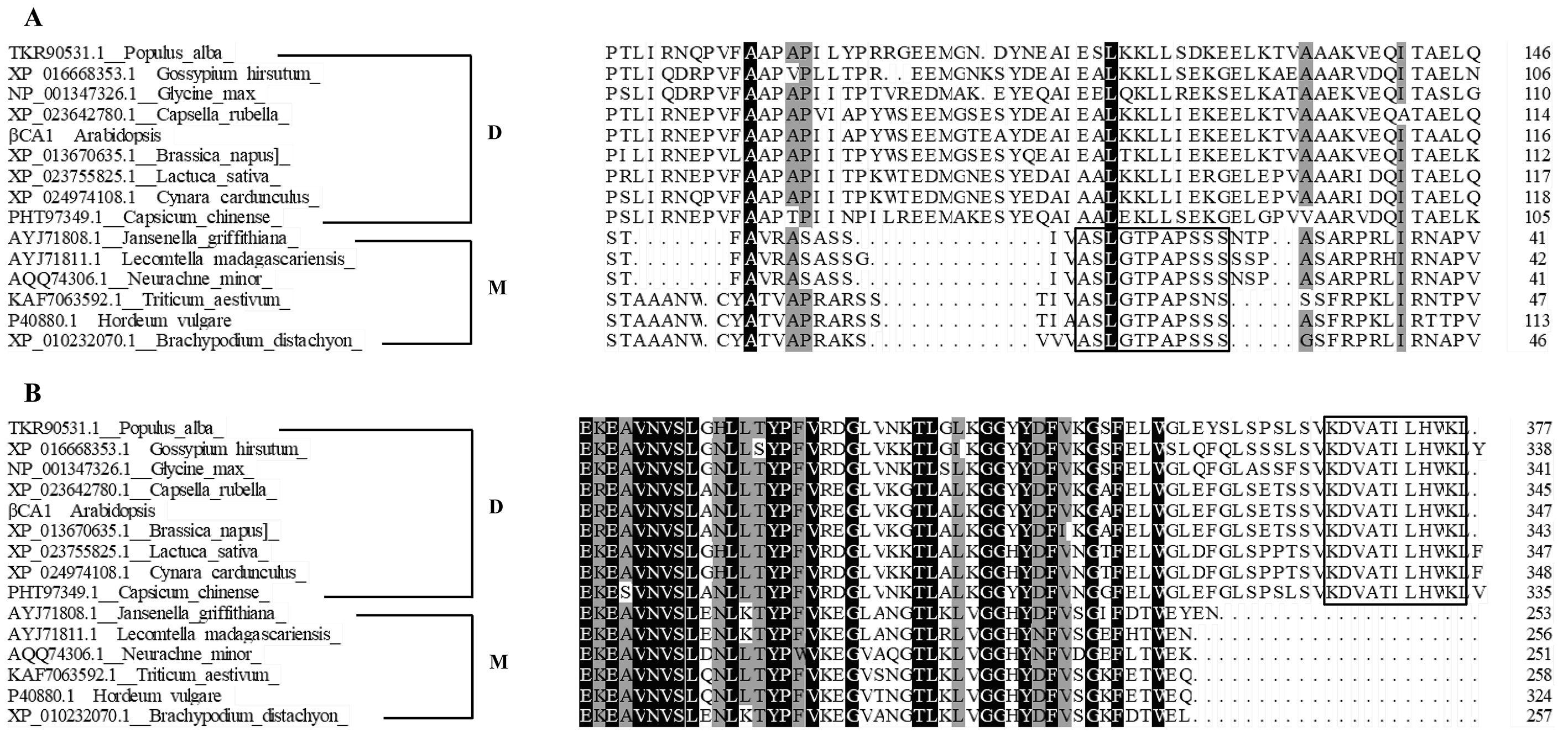

Supplement: Supplemental Information 6 — The specific short peptides required for chloroplast targeting were heighted in black boxes. D: dicot; M: monocot. [file peerj-09-12673-s006.jpg]

Figure 1C

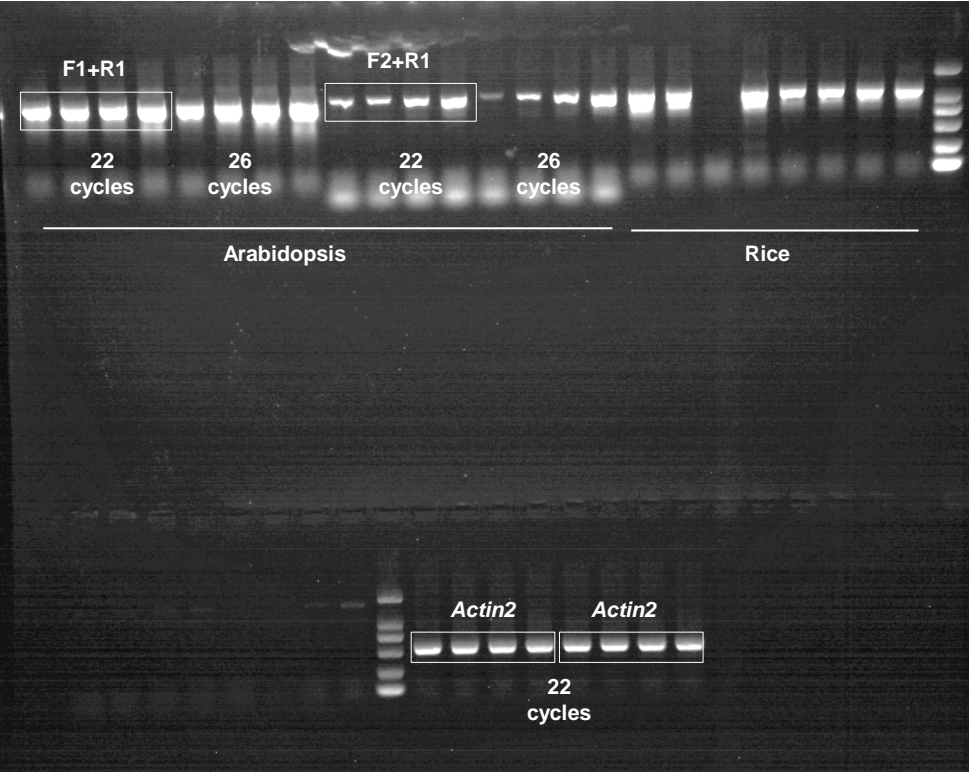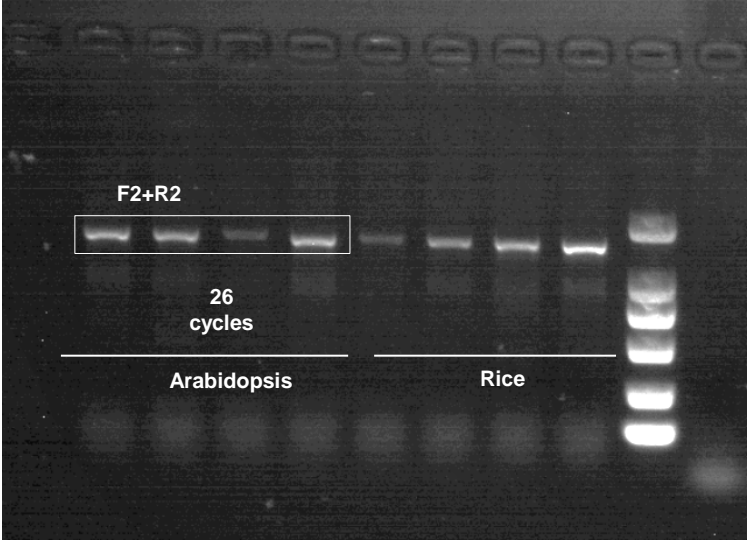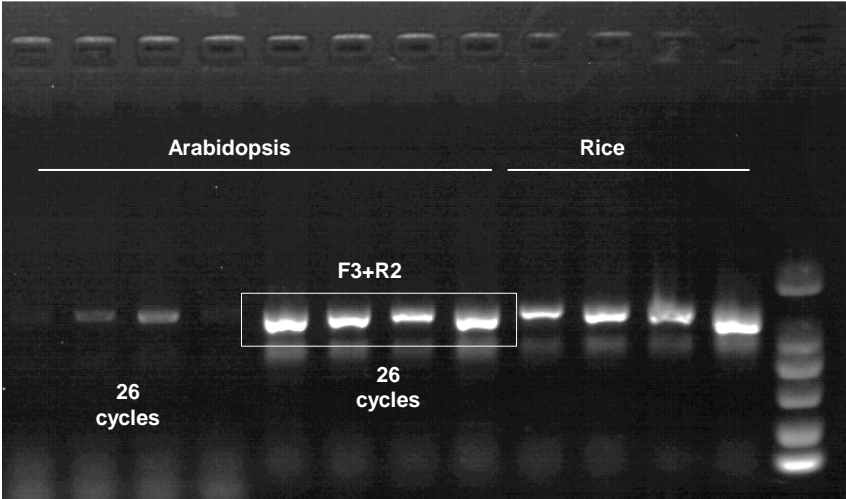

Figure 1D

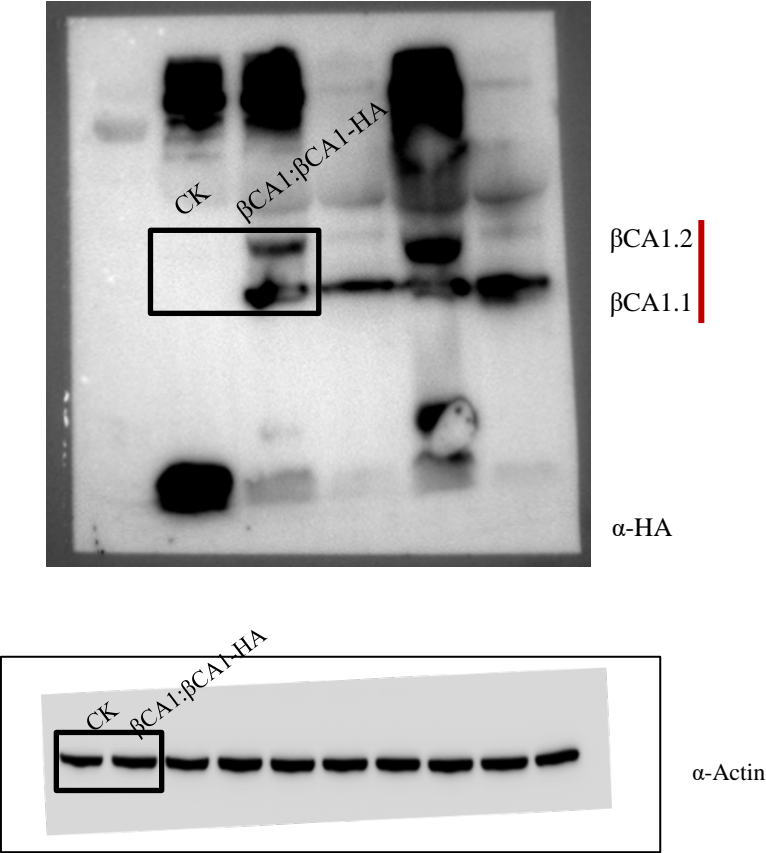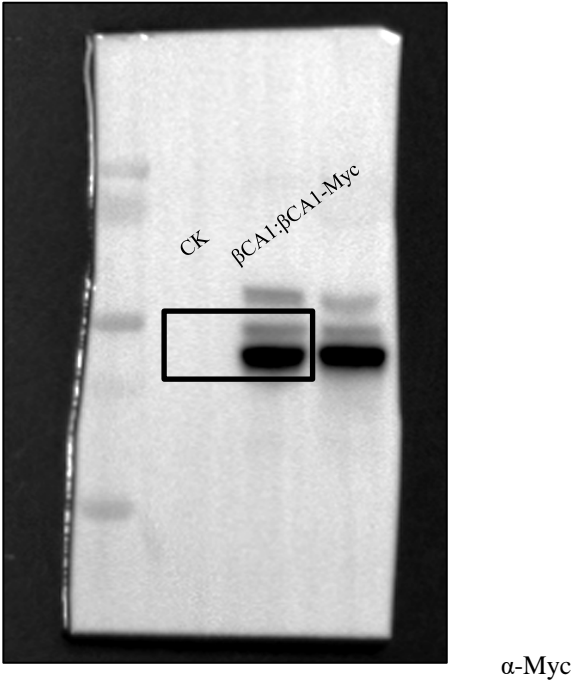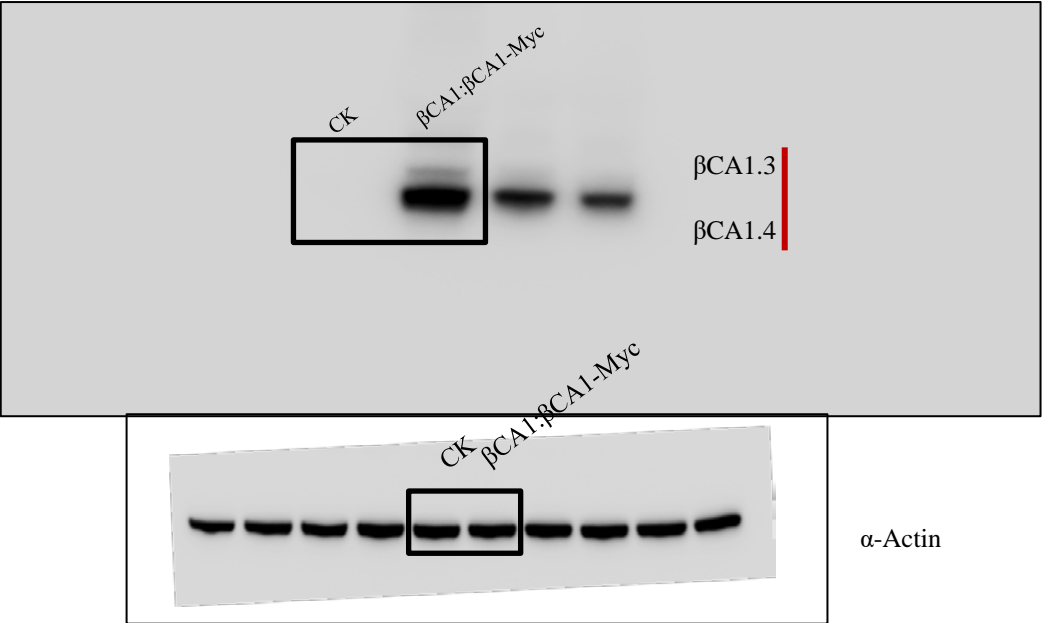

Figure 3A

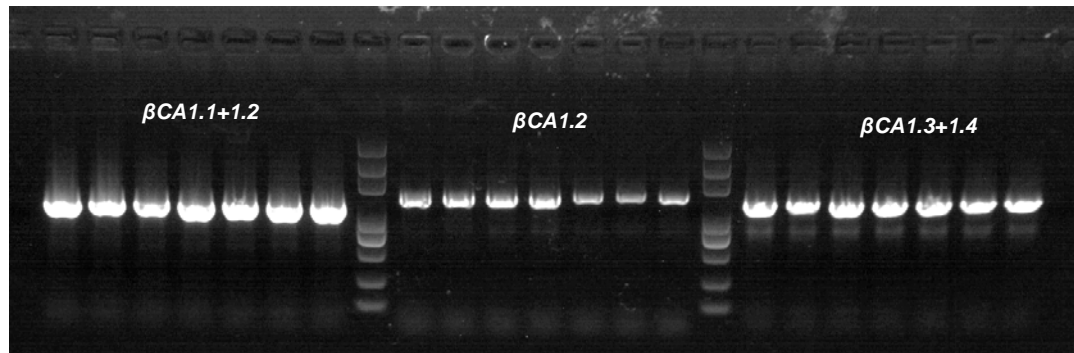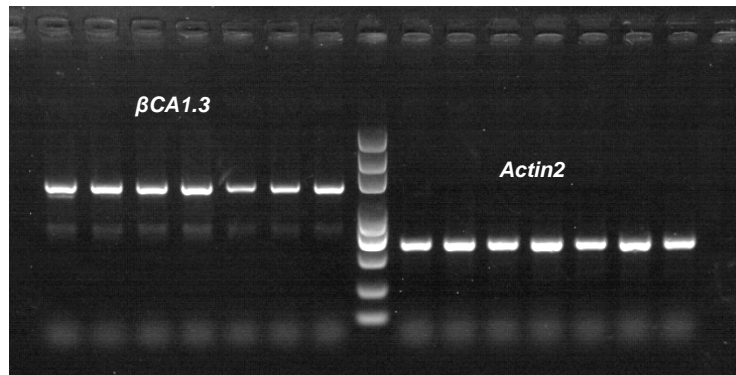

Supplement: Supplemental Information 7 [file peerj-09-12673-s007.pdf]
